# Free fatty acid binding pocket in the locked structure of SARS-CoV-2 spike protein

**DOI:** 10.1126/science.abd3255

**Published:** 2020-09-21

**Authors:** Christine Toelzer, Kapil Gupta, Sathish K. N. Yadav, Ufuk Borucu, Andrew D. Davidson, Maia Kavanagh Williamson, Deborah K. Shoemark, Frederic Garzoni, Oskar Staufer, Rachel Milligan, Julien Capin, Adrian J. Mulholland, Joachim Spatz, Daniel Fitzgerald, Imre Berger, Christiane Schaffitzel

**Affiliations:** 1 School of Biochemistry, University of Bristol, 1 Tankard’s Close, Bristol BS8 1TD, UK.; 2 Bristol Synthetic Biology Centre BrisSynBio, 24 Tyndall Ave., Bristol BS8 1TQ, UK.; 3 School of Cellular and Molecular Medicine, University of Bristol, University Walk, Bristol BS8 1TD, UK.; 4 Imophoron Ltd., St. Philips Central, Albert Rd., St. Philips, Bristol BS2 0XJ, UK.; 5 Department for Cellular Biophysics, Max Planck Institute for Medical Research, Jahnstraße 29, 69120 Heidelberg, Germany.; 6 Institute for Physical Chemistry, Department for Biophysical Chemistry, University of Heidelberg, Im Neuenheimer Feld 253, 69120 Heidelberg, Germany.; 7 Max Planck School Matter to Life, Jahnstraße 29, D-69120 Heidelberg, Germany.; 8 Max Planck Bristol Centre for Minimal Biology, Cantock’s Close, Bristol BS8 1TS, UK.; 9 School of Chemistry, University of Bristol, Cantock’s Close, Bristol BS8 1TS, UK.; 10 Geneva Biotech Sàrl, Avenue de la Roseraie 64, 1205, Geneva, Switzerland.

## Abstract

Coronavirus disease 2019 (COVID-19), caused by severe acute respiratory syndrome coronavirus 2 (SARS-CoV-2), represents a global crisis. Key to SARS-CoV-2 therapeutic development is unraveling the mechanisms that drive high infectivity, broad tissue tropism, and severe pathology. Our 2.85-angstrom cryo–electron microscopy structure of SARS-CoV-2 spike (S) glycoprotein reveals that the receptor binding domains tightly bind the essential free fatty acid linoleic acid (LA) in three composite binding pockets. A similar pocket also appears to be present in the highly pathogenic severe acute respiratory syndrome coronavirus (SARS-CoV) and Middle East respiratory syndrome coronavirus (MERS-CoV). LA binding stabilizes a locked S conformation, resulting in reduced angiotensin-converting enzyme 2 (ACE2) interaction in vitro*.* In human cells, LA supplementation synergizes with the COVID-19 drug remdesivir, suppressing SARS-CoV-2 replication. Our structure directly links LA and S, setting the stage for intervention strategies that target LA binding by SARS-CoV-2.

## Locking down the SARS-CoV-2 spike

Many efforts to develop therapies against severe acute respiratory syndrome coronavirus 2 (SARS-CoV-2) are focused on the spike (S) protein trimer that binds to the host receptor. Structures of trimeric S protein show its receptor-binding domain in either an up or a down conformation. Toelzer *et al.* produced SARS-CoV-2 S in insect cells and determined the structure by cryo–electron microscopy. In their dataset, the closed form was predominant and was stabilized by binding linoleic acid, an essential fatty acid. A similar binding pocket appears to be present in previous highly pathogenic coronaviruses, and past studies suggested links between viral infection and fatty acid metabolism. The pocket could be exploited to develop inhibitors that trap S protein in the closed conformation.

*Science*, this issue p. 725

## Teaser

The SARS-CoV-2 spike binds linoleic acid, a key molecule in inflammation, immune modulation, and membrane fluidity.

Seven coronaviruses are known to infect humans. The four endemic human coronaviruses (HCoVs)—OC43, 229E, HKU1, and NL63—cause mild upper respiratory tract infections, whereas pandemic virus SARS-CoV-2 (severe acute respiratory syndrome coronavirus 2) and earlier SARS-CoV (severe acute respiratory syndrome coronavirus) and MERS-CoV (Middle East respiratory syndrome coronavirus) can cause severe pneumonia with acute respiratory distress syndrome, multi-organ failure, and death (*1*, *2*).

SARS-CoV-2 has acquired functions that promote its harsh disease phenotype. SARS-CoV-2 causes severe inflammation and damage to endothelial cells in the heart, kidneys, liver, and intestines, suggestive of a vascular infection rather than a purely respiratory disease (*3*, *4*). The attachment of SARS-CoV-2 to a host cell is initiated by the spike (S) protein trimer, which adorns the outer surface of the virus, binding to its cognate receptor angiotensin-converting enzyme 2 (ACE2) with higher affinity than the SARS-CoV S protein (*5*–*7*). A S1-S2 polybasic furin protease cleavage site distinguishes SARS-CoV-2 from SARS-CoV and other closely related bat coronaviruses and serves to stimulate entry into host cells and cell-cell fusion (*5*, *8*, *9*). Inside the host cell, HCoVs remodel the lipid metabolism to facilitate virus replication (*10*). Infection by SARS-CoV-2 triggers an unusually impaired and dysregulated immune response (*11*) and a heightened inflammatory response (*12*), working in synergy with interferon production in the vicinity of infected cells to drive a feed-forward loop to up-regulate ACE2 and further escalate infection (*13*).

**Fig. 1:**
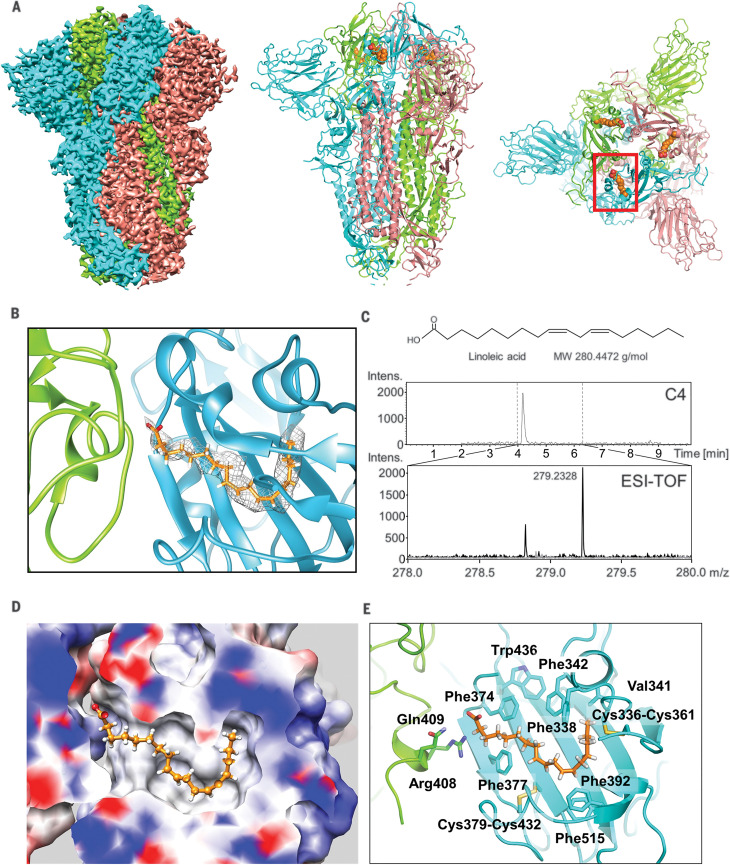
Cryo-EM structure of the SARS-CoV-2 S linoleic acid complex. (**A**) Cryo-EM density of the S trimer is shown (left). Monomers are in cyan, green, and pink, respectively. The structure is also shown in a cartoon representation in a front view (middle) and top view (right). Bound LA is illustrated as orange spheres. One LA binding pocket is surrounded by the red box. (**B**) Composite LA binding pocket formed by adjacent RBDs. Tube-shaped EM density is shown. (**C**) LC-MS analysis of purified S. (Top) Chemical structure and molecular weight (MW) of LA. (Middle) C4 column elution profile. (Bottom) Electrospray ionization time-of-flight (ESI-TOF) spectra of wash solution (gray) and C4 peak elution fraction (black), with peak molecular weight indicated. Intens., intensity, m/z, mass/charge ratio. (**D**) Hydrophobic LA binding pocket in a surface representation, illustrating the excellent fit of bound LA (orange; shown in ball-and-stick representation). Blue and red indicate positive and negative surface charge, respectively. (**E**) LA interactions with amino acids in the binding pocket. The acidic LA headgroup is in the vicinity of an arginine (Arg^408^) and a glutamine (Gln^409^).

In the search for additional functions that contribute to the pathology of infection, we determined the structure of the SARS-CoV-2 S glycoprotein by cryo–electron microscopy (cryo-EM) (Fig. 1). We produced SARS-CoV-2 S as a secreted trimer (*14*) in MultiBac (*15*) baculovirus-infected Hi5 insect cells (fig. S1) (*16*). Highly purified protein was used for cryo-EM data collection (fig. S2 and table S1). After three-dimensional (3D) classification and refinement without applying symmetry (C1), we obtained a 3.0-Å closed conformation from 136,405 particles and a 3.5-Å open conformation with one receptor binding domain (RBD) in the up position from 57,990 particles (figs. S2 and S3). C3 symmetry was applied to the closed conformation particle pool, yielding a 2.85-Å map (Fig. 1A and figs. S2 and S3).

The structure of S displays the characteristic overall shape observed for coronavirus S proteins in the closed and open conformations (*17*–*19*), with the closed form (~70%) predominating in our dataset (Fig. 1A and figs. S2 to S4). Model building of the closed form revealed additional density in the RBDs in our structure (Fig. 1B). The tubelike shape of this density was consistent with a fatty acid, with size and shape similar to that of linoleic acid (LA) bound to other proteins (Fig. 1B and fig. S5) (*20*, *21*). Liquid chromatography–coupled electrospray ionization time-of-flight mass spectrometry (LC-MS) analysis confirmed the presence of a compound with the molecular weight of LA in our purified sample (Fig. 1C).

The hallmarks of free fatty acid (FFA) binding pockets in proteins are an extended “greasy” tube lined by hydrophobic amino acids, which accommodates the hydrocarbon tail, and a hydrophilic, often positively charged anchor for the acidic headgroup of the FFA. In our structure, a hydrophobic pocket mostly shaped by phenylalanines forms a bent tube into which the LA fits well (Fig. 1, D and E). The anchor for the headgroup carboxyl is provided by an arginine (Arg^408^) and a glutamine (Gln^409^) from the adjacent RBD in the trimer, giving rise to a composite LA binding site (Fig. 1E). We confirmed the presence of LA in all three binding pockets in the S trimer in the unsymmetrized (C1) closed structure (fig. S6). Similarly, masked 3D classification focusing on the RBDs could not identify any unoccupied pockets (fig. S7).

**Fig. 2:**
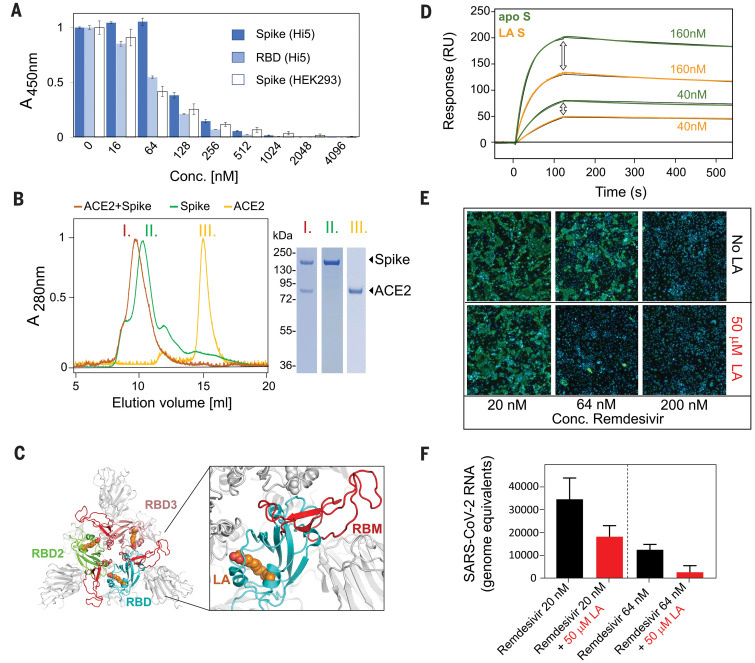
Functional characterization of LA-bound SARS-CoV-2 S. (**A**) Insect cell (Hi5)–expressed S (dark blue bars), insect cell–expressed RBD (light blue bars), and mammalian (HEK293)–expressed S (white bars) in competition ELISAs with immobilized ACE2. Error bars indicate SDs (three replicates). A, absorbance; Conc., concentration. (**B**) Interaction of LA-bound SARS-CoV-2 S protein with ACE2 was analyzed by SEC, evidencing complex formation. (Left) SEC profiles are shown for ACE2 (yellow, III), LA-bound S (green, II), and a mixture of ACE2 and LA-bound S (orange, I). (Right) Peak fractions (I to III) were analyzed by SDS–polyacrylamide gel electrophoresis, evidencing the expected proteins. (**C**) (Left) Top view of the LA-bound S glycoprotein trimer, with RBDs shown in cyan, green, and pink. In each RBD subunit, the motif responsible for ACE2 binding (RBM) is in red, and LA is shown as orange spheres. (Right) A close-up view into the cyan RBD shows that the RBM is fully ordered and that LA and RBM are not in direct contact. (**D**) SPR analysis of binding of the LA-bound S trimer (orange curves) and the apo S trimer (green curves) to immobilized ACE2. Apo S and LA-bound S were diluted to concentrations of 40 and 160 nM, respectively. Black lines correspond to a global fit from a 1:1 binding model. RU, resonance units. (**E**) Synergistic effect of LA and remdesivir on SARS-CoV-2 replication. Effects of varying doses of remdesivir ± 50 μM LA on virus infection are shown. Human Caco-2 ACE2+ cells were infected with SARS-CoV-2 and then treated with varying doses of remdesivir ± 50 μM LA. At 96 hours after infection, cells were fixed and infected cells were detected by immunofluorescence assay using an anti-N antibody (green). Cell nuclei were stained by 4′,6-diamidino-2-phenylindole (DAPI; blue). Representative images corresponding to the remdesivir dose range of 20 to 200 nM are shown. (**F**) Amount of extracellular virus present in wells (*n* = 3) at the dose combinations shown was determined by quantitative reverse transcription polymerase chain reaction (performed in duplicate for each sample). Error bars show SD.

Our S construct contains alterations relative to native SARS-CoV-2 S—namely, addition of a trimerization domain and deletion of the polybasic cleavage site, neither of which alters the S conformation appreciably (*14*, *17*) (fig. S8). Glycosylation sites are located away from the LA binding pocket and are largely native in our structure (*7*, *17*) (table S2). Thus, neither glycosylation nor mutations are likely to affect the LA binding pocket. We compared S and RBD produced in insect cells with mammalian-produced S to identify any potential influence of differences in glycosylation on ACE2 binding by competition enzyme-linked immunosorbent assay (ELISA) (Fig. 2A). All three reagents bound ACE2 efficiently. We then used size exclusion chromatography (SEC) with purified proteins to further confirm ACE2 binding by S (Fig. 2B). The LA binding pocket and the receptor binding motif (RBM) are distal and nonoverlapping (Fig. 2C). Notably, in the LA-bound S, the RBM is ordered and buried at the interface between RBDs, whereas it was disordered in previously described SARS-CoV-2 S cryo-EM structures (*7*, *17*).

SARS-CoV-2 S can also adopt an open conformation (fig. S4), which is compatible with binding ACE2. In previous apo S cryo-EM structures, about 60 to 75% of the S trimers were in the open conformation (*7*, *17*), in contrast to our observation of ~70% in the closed conformation. This result could be due to LA stabilizing the closed conformation; if so, LA would be expected to reduce ACE2 binding. We performed surface plasmon resonance (SPR) experiments with biotinylated ACE2 immobilized on a streptavidin-coated chip (Fig. 2D and fig. S9). We first determined the dissociation constant (*K*_D_) of the RBD-ACE2 interaction to validate our assay. Our value (26 nM; fig. S9C) is in good agreement with that of previous work [44 nM (*22*)], which used SPR with the RBD immobilized and ACE2 as an analyte. Apo S was prepared by applying Lipidex, the established method for removing lipids from lipid-binding proteins (*23*). A *K*_D_ of 0.7 nM was obtained for the apo S-ACE2 interaction (fig. S9A). For the LA-bound S-ACE2 interaction, we obtained a *K*_D_ of 1.4 nM (fig. S9B). We consistently obtained a markedly reduced resonance unit signal for LA-bound S compared with apo S at the same concentrations (Fig. 2D and fig. S9, A and B). This observation is consistent with the apo state having a higher percentage of S trimers in the open, ACE2-accessible conformation.

We characterized the affinity of the LA interaction both experimentally and computationally. Our SPR assays with immobilized RBD yielded a binding constant of ~41 nM, exhibiting a slow off-rate, consistent with tight binding of LA (fig. S10). Repeated molecular dynamics simulations of the entire locked LA-bound S trimer (three 100-ns simulations) using GROMACS-2019 (*24*) corroborated the persistence of stable interactions between LA and the S trimer (movies S1 and S2). The affinity of LA binding to the S trimer will likely be higher than that of binding to the RBD alone, taking into account polar headgroup interactions with Arg^408^ and Gln^409^ of the adjacent RBD (Fig. 1E). The resolution of the RBDs in our open S cryo-EM structure was insufficient to either assign or rule out a ligand-bound pocket (fig. S3). However, the slow off-rate observed with the RBD monomer (fig. S10) suggests that LA binding could be maintained when the S trimer transiently converts into the open conformation. This is supported by our observation that LA was retained during S purification, in spite of S trimers adopting the open form ~30% of the time (fig. S2), and by our MD simulations with a modeled ligand-bound open S trimer (movie S3) in which all three LAs remained bound for 500 ns.

Next, we investigated the effect of LA in experiments with live SARS-CoV-2 infecting human epithelial cells. Remdesivir is an RNA-dependent RNA polymerase inhibitor and the first antiviral drug to show a benefit in the treatment of coronavirus disease 2019 (COVID-19) in clinical trials, albeit with considerable side effects at the doses required (*25*). LA supplementation at concentrations of 50 to 100 μM was previously shown to affect coronavirus entry and replication (*10*). We administered remdesivir at concentrations of 20, 64, and 200 nM, supplementing with 50 μM LA (Fig. 2E). Our results revealed synergy, with the dose of remdesivir required to suppress SARS-CoV-2 replication markedly reduced by the addition of LA (Fig. 2, E and F).

**Fig. 3:**
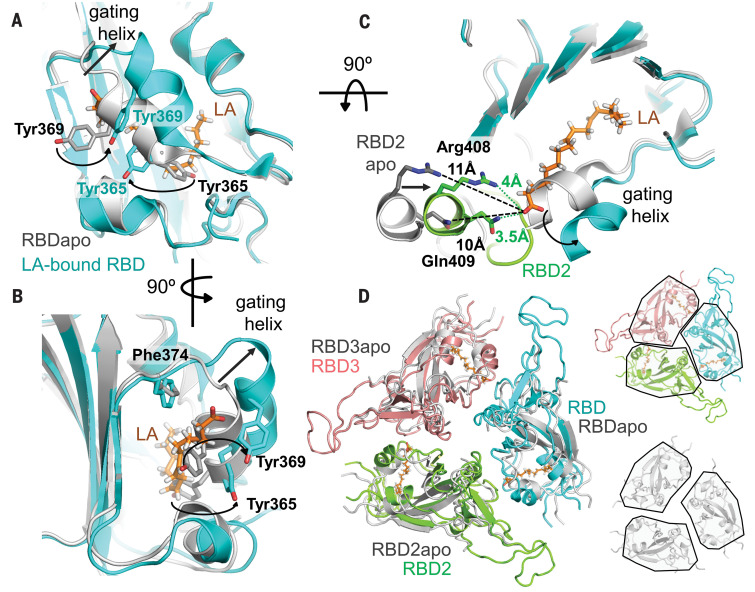
Comparison of LA-bound and apo S structures. (**A**) Superimposition of LA-bound SARS-CoV-2 RBD (cyan) and ligand-free apo RBD (gray) [PDB ID 6VXX (*7*)]. The gating helix at the entrance of the hydrophobic pocket moves by 6 Å in the presence of LA. Tyr^365^ and Tyr^369^ swing away, avoiding clashes with LA (orange). Black arrows indicate the rearrangements. (**B**) Same structure as in (A) rotated by 90° as indicated, showing the entrance of the hydrophobic pocket. (**C**) Formation of a composite LA binding pocket by two adjacent RBDs in LA-bound S involves a ~5-Å movement of RBD2 (green) toward RBD1 (cyan) as opposed to apo S (gray). (**D**) Superimposition of the RBD trimer of apo S (gray) and LA-bound S (RBD1, cyan; RBD2, green; RBD3, pink; LA, orange) is shown (left). The individual RBD trimers are depicted for LA-bound S (right, top) and apo S (right, bottom), with RBDs boxed in black, highlighting the compaction of RBDs in the LA-bound S structure.

We superimposed our LA-bound structure on previous SARS-CoV-2 apo S structures in the closed conformation (*7*, *17*) and identified a gating helix located directly at the entrance of the binding pocket (Fig. 3, A to C). This gating helix, which comprises Tyr^365^ and Tyr^369^, is displaced by ~6 Å when LA is bound, thus opening the pocket (Fig. 3, A and B). In the apo SARS-CoV-2 S trimer (*7*, *17*), a gap between adjacent RBDs places the hydrophilic anchor residues ~10 Å from the position of the LA headgroup (Fig. 3C). Upon LA binding, the adjacent RBD in the trimer moves toward its neighbor, and the anchor residues Arg^408^ and Gln^409^ lock down on the headgroup of LA (Fig. 3, C and D). Overall, this results in a compaction of trimer architecture in the region formed by the three RBDs, producing a locked S structure (Fig. 3D and movie S4).

**Fig. 4:**
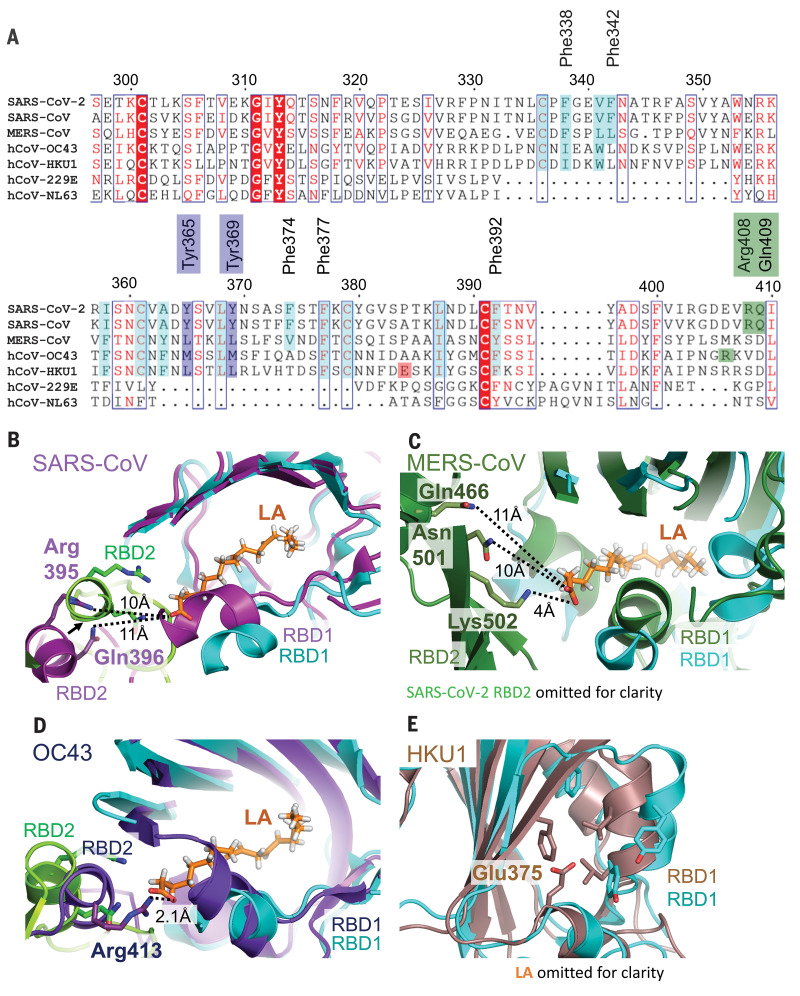
Human coronavirus RBD architectures. (**A**) Alignments of the seven CoV strains that can infect humans, highlighting conserved residues. Cyan, residues lining the hydrophobic pocket; purple, gating helix residues; green, residues positioned to interact with the LA polar headgroup; and red, Glu^375^ in HKU1 [see panel (E)]. Single-letter abbreviations for the amino acid residues are as follows: A, Ala; C, Cys; D, Asp; E, Glu; F, Phe; G, Gly; H, His; I, Ile; K, Lys; L, Leu; M, Met; N, Asn; P, Pro; Q, Gln; R, Arg; S, Ser; T, Thr; V, Val; W, Trp; and Y, Tyr. (**B**) Superimposition of RBD1 of LA-bound SARS-CoV-2 (RBD2, green) with RBD1 of ligand-free apo SARS-CoV [RBD1 and RBD2, magenta; PDB ID 5X58 (*19*)] indicates a conservation of the composite binding pocket. (**C**) Superimposition of RBD1 of LA-bound SARS-CoV-2 (RBD2 is omitted for clarity) with RBD1 of MERS-CoV [RBD1 and RBD2, forest green; PDB ID 5X5F (*19*)]. (**D**) Superimposition of RBD1 of LA-bound SARS-CoV-2 (RBD2, green) with RBD1 of OC43 [RBD1 and RBD2, purple; PDB ID 6NZK (*18*)]. (**E**) Superimposition of LA-bound SARS-CoV-2 RBD with HKU1 RBD [brown; PDBID 5GNB (*26*)]. LA is omitted in SARS-CoV-2 RBD for clarity.

We investigated whether the LA-binding pocket is conserved in the seven coronaviruses that infect humans (Fig. 4A and table S3). Sequence alignment shows that all residues lining the hydrophobic pocket and the anchor residues (Arg^408^ and Gln^409^) in SARS-CoV-2 are fully conserved in SARS-CoV (Fig. 4A). Structural alignment of LA-bound RBDs within the trimer of SARS-CoV-2 and apo SARS-CoV RBDs (*19*) reveals that the LA binding pocket is present in SARS-CoV. The greasy tube is flanked by a gating helix as in SARS-CoV-2, with Arg^395^ and Gln^396^ of SARS-CoV positioned 10 and 11 Å from the entrance, respectively, in a conformation that is virtually identical to that of apo SARS-CoV-2 (Figs. 3C and 4B). In MERS-CoV, the gating helix and hydrophobic residues lining the pocket are also present. Tyr^365^, Tyr^369^, and Phe^374^ are substituted by likewise hydrophobic leucines and a valine, respectively (Fig. 4, A and C) (*19*). The Arg^408^-Gln^409^ pair is not conserved; however, we identify Asn^501^, Lys^502^, and Gln^466^ as potential anchor residues, located on a β sheet and an α helix within the adjacent RBD, up to 11 Å from the entrance (Fig. 4C). Thus, the greasy tube and hydrophilic anchor appear to be present in MERS-CoV, suggesting convergent evolution. In HCoV OC43, the gating helix and hydrophobic residues that line the pocket are largely conserved, whereas Tyr^365^, Tyr^369^, and Phe^374^ are replaced by methionines and alanine, respectively (Fig. 4A) (*18*). Arg^413^ is located on the same helix as Arg^408^ and Gln^409^ in SARS-CoV-2 and could serve as a hydrophilic anchor (Fig. 4D). No gap exists in this presumed apo form structure between the RBDs, which appear already in the locked conformation (Fig. 4D and fig. S11) (*18*). In HCoV HKU1, the hydrophobic residues are again largely conserved, but a charged residue (Glu^375^) is positioned directly in front of the entrance, obstructing access for a putative ligand (Fig. 4E) (*26*). The RBDs of HCoVs 229E and NL63 adopt a very different fold (fig. S13) (*27*, *28*), and many of the LA binding residues are not present (Fig. 4A), thus hampering predictions of a binding site for fatty acids.

In summary, we find four molecular features that mediate LA binding to SARS-CoV-2 and potentially also to SARS-CoV and MERS-CoV S proteins: a conserved hydrophobic pocket, a gating helix, amino acid residues prepositioned to interact with the LA carboxyl headgroup, and loosely packed RBDs in the apo form. By contrast, in each of the four common circulating HCoVs, it appears that one or more of these four architectural prerequisites is lacking in the S protein structures (Fig. 4 and figs. S11 and S12). LA binding to SARS-CoV-2 S triggers a locking down of the hydrophilic anchor and a compaction of the RBD trimer (Fig. 3, C and D). In addition to stabilizing the closed conformation, this lockdown could also help stabilize the S1 region, which includes the N-terminal domain and the RBD. The RBM, central to ACE2 binding, appears to be conformationally preorganized in our structure (Fig. 2C), indicating a generally more rigid RBD trimer when LA is bound. Although direct cross-talk between the LA binding pocket and the RBM is not apparent from our structure (Fig. 2C), the conformational changes in the RBD trimer (Fig. 3) could affect ACE2 docking and infectivity, as indicated by our SPR assays that show reduced levels of S binding in the presence of LA (Fig. 2D). The S protein’s tight binding of LA originates from a well-defined size and shape complementarity afforded by the pocket (Fig. 1, B and D). The LA binding pocket thus presents a promising target for future development of small-molecule inhibitors that, for example, could irreversibly lock S in the closed conformation and interfere with receptor interactions. It is noteworthy in this context that a fatty acid binding pocket was exploited previously to develop potent small-molecule antiviral drugs to treat rhinovirus, locking viral surface proteins in a conformation incompatible with receptor binding (*29*, *30*). These antivirals were successful in human clinical trials (*31*, *32*).

A recent proteomic and metabolomic study of COVID-19 patient sera showed continuous decrease of FFAs, including LA (*33*). Lipid metabolome remodeling is a common element of viral infection (*34*, *35*). For coronaviruses, the LA–to–arachidonic acid metabolic pathway was identified as central to lipid remodeling (*10*). We hypothesize that LA sequestration by SARS-CoV-2 could confer a tissue-independent mechanism by which pathogenic coronavirus infection may drive immune dysregulation and inflammation (*35*–*37*). Our findings provide a direct structural link between LA, COVID-19 pathology, and the virus itself and suggest that both the LA binding pocket within the S protein and the multinodal LA signaling axis represent excellent therapeutic intervention points to treat SARS-CoV-2 infections.

## Supplementary Materials

science.sciencemag.org/content/370/6517/725/suppl/DC1 Materials and Methods Figs. S1 to S12 Tables S1 to S3 References (*41*–*63*) MDAR Reproducibility Checklist Movies S1 to S4

## Appendix

View/request a protocol for this paper from *Bio-protocol*.
